# Delamination Fracture Behavior of Unidirectional Carbon Reinforced Composites Applied to Wind Turbine Blades

**DOI:** 10.3390/ma14030593

**Published:** 2021-01-27

**Authors:** Ana Boyano, Jose Manuel Lopez-Guede, Leyre Torre-Tojal, Unai Fernandez-Gamiz, Ekaitz Zulueta, Faustino Mujika

**Affiliations:** 1 Mechanical Engineering Department, Faculty of Engineering of Vitoria-Gasteiz, University of the Basque Country, UPV/EHU, Nieves Cano 12, 01006 Vitoria-Gasteiz, Spain; ana.boyano@ehu.eus; 2 Systems Engineering and Automation Control Department, Faculty of Engineering of Vitoria-Gasteiz, University of the Basque Country, UPV/EHU, Nieves Cano 12, 01006 Vitoria-Gasteiz, Spain; ekaitz.zulueta@ehu.es; 3 Mining and Metallurgical Engineering and Materials Science Department, Faculty of Engineering of Vitoria-Gasteiz, University of the Basque Country, UPV/EHU, Nieves Cano 12, 01006 Vitoria-Gasteiz, Spain; leyre.torre@ehu.eus; 4 Nuclear Engineering and Fluid Mechanics Department, Faculty of Engineering of Vitoria-Gasteiz, University of the Basque Country, UPV/EHU, Nieves Cano 12, 01006 Vitoria-Gasteiz, Spain; unai.fernandez@ehu.eus; 5 Mechanical Engineering Department, Faculty of Engineering Gipuzkoa, University of the Basque Country, UPV/EHU, Plaza Europa 1, 20018 Donostia-San Sebastián, Spain; faustino.mujika@ehu.eus

**Keywords:** wind turbine blades, delamination, fracture tests

## Abstract

One of the materials that is used widely for wind turbine blade manufacturing are fiber-reinforced composites. Although glass fiber reinforcement is the most used in wind turbine blades, the use of carbon fiber allows larger blades to be manufactured due to their better mechanical characteristics. Some turbine manufacturers are using carbon fiber in the most critical parts of the blade design. The larger rotors are exposed to complex loading conditions in service. One of the most relevant structures on a wind turbine blade is the spar cap. It is usually manufactured by means of unidirectional laminates, and one of its major failures is the delamination. The determination of material features that influence delamination initiation and advance by appropriate testing is a fundamental topic for the study of composite delamination. The fracture behavior is studied across coupons of carbon fiber reinforcement epoxy laminates. Fifteen different test conditions have been analyzed. Fracture surfaces for different mode ratios have been explored using optical microscope and scanning electron microscope. Experimental results shown in the paper for critical fracture parameters agree with the theoretically expected values. Therefore, this experimental procedure is suitable for wind turbine blade material characterizing at the initial coupon-scale research level.

## 1. Introduction

Wind energy generation is a fundamental issue within energy production in order to reduce fossil fuel necessity. In the last years, the number and size of the wind energy farms have increased. For instance, in the European Union (EU), wind based production is expected to be over 200 GW and become the largest renewable source of energy [1]. The development of new wind turbines will tend toward the design of larger ones and offshore wind farms. The durability of large wind turbines is particularly crucial due to the high wind loads and the high cost of repair. The optimum service life for a wind turbine is about 20 years, and the repair or maintenance is expected to be minimum. Therefore, one of the challenges of wind energy is to minimize the maintenance and repair costs [2].

Optimum features of materials suitable for wind turbines are: high stiffness, low weight and their ability to withstand fatigue damage. Composite materials are usually designed to have these properties. The characteristics of the composite material of blades often establish the performance and lifetime of the wind turbine. Rotor blades are often made from different subcomponents of fiber-reinforced composites linked together [3]. Blades are primarily manufactured by fiberglass composite. However, the increase of the rotor size has led to the consideration of carbon fiber, at least in some parts of the blade [4,5,6,7]. Due to their specific features, carbon fiber reinforced polymers have been broadly investigated in many different sectors, such as building structures [8,9], automotive sector [10,11,12] or energy harvesting applications [13,14,15], among others. Carbon fiber has lower density and higher specific strength and stiffness. The disadvantage is the higher cost, but this drawback could be minimized in the case of the wind energy, by reducing the blade weight [16].

**Figure 1:**
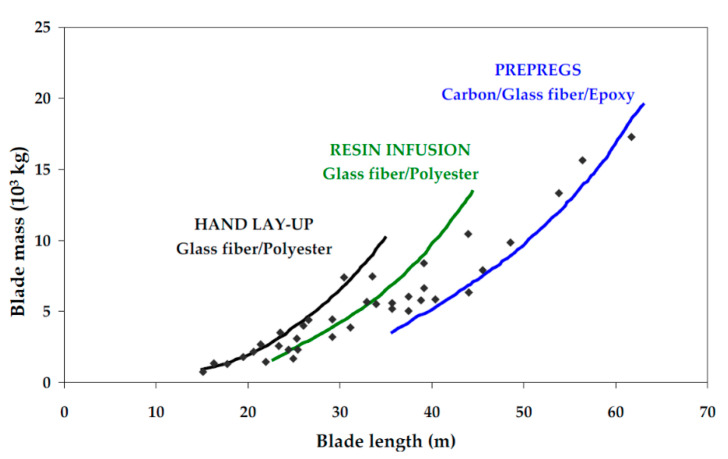
Manufacturing process and materials used in terms of blade mass and blade length [17].

Regarding the manufacturing process, using prepregs instead of resin infusion has the advantage of giving better composite material properties and, again, the disadvantage of the higher cost. However, it is a technological step for the mass reduction of the blades when manufacturing bigger blades, as is shown in Figure 1 [17].

Delamination between layers is one of the principal causes of many problems of structures made by composite material, among others, wind turbine blades [18]. Separation between adjacent plies (delamination) is one of the most common failure processes in laminates, because of the low through-thickness strength of laminates. Conclusions obtained from different experimental investigations on structural tests show that the blade collapse was caused, due to a number of failure modes observed, by the delamination of unidirectional laminates in the spar cap [19]. In an investigation at structural level of wind turbines damaged during the super typhoon Usagi (2013), tower collapse and blade fracture were the major structural failures found in wind turbines. In other remarks, authors found that the most likely origin of the blade fracture was that the blade strain exceeded the failure strain of unidirectional composites in the spar cap [20]. In some failure analyses of a 52.3 m composite wind turbine blade, it was found that accumulated delamination of unidirectional composites in the spar cap was one of the main reason for the blade collapse [21,22]. Delamination often takes place due to manufacturing process or in-service loads, or even due to water droplet erosion [23]. Hence, delamination characterization is essential to study the damage tolerance in composite structures.

Delamination in a skin-stiffener intersection was investigated by Mandell et al. [24] for the case of a wind turbine blade. Authors used methodologies based on fracture mechanics in order to determine the critical strain energy release rate to select the proper material. Their main conclusion was that resins that produce improved interlaminar toughness led to greater resistance against damage development. The cohesive zone model approach, which is based also on fracture mechanics, can be used in damage analysis [25].

The development of new composite blades should involve an interaction between materials, the manufacturing process and structural design.

**Figure 2:**
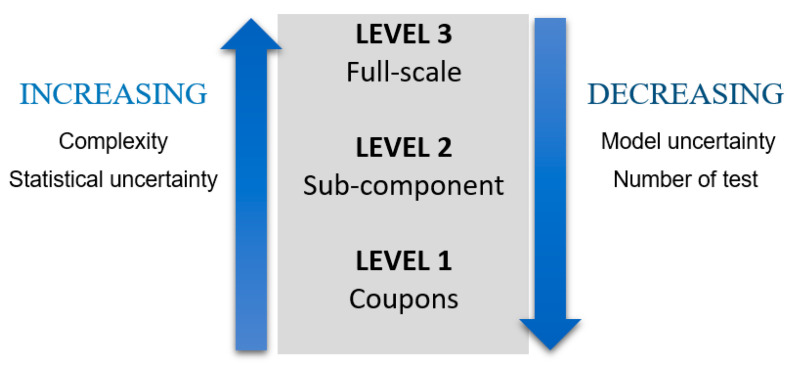
Different test scales [26].

Regarding materials’ development, some key issues include improvement of material properties or better understanding of material behavior. Experimental tests can be carried out on different scales to determine relevant material properties and to assess the numerical models used to simulate loading effects. In Figure 2 types of tests depending on the scale are shown. Nevertheless, to certify a wind turbine blade, according to the IEC 61400 standard, only coupon test and full-scale test are required [26,27].

In order to provide a better description of the materials’ properties, better material models and testing methods are needed [28].

Nowadays it is still an open field and a challenging task to design and implement scaled models of structures made of composite materials, since scaling the thickness of the plies of a laminate has practical difficulties. Thus, it is hard to implement composite models with scaled-down size having a completely similar lamination scheme as the prototype to analyze. Thereupon, using partially similar scaled models can be considered as an a feasible alternative [27], and this could be a future work. Once coupons and subcomponents having a processing route and a representative lay-up similar to the blade to study, mechanical tests can be carried out on them. The size effect of the laboratory scale test is an important issue in these cases. Salviato et al. [29] analyzed Bažant’s size effect law to take into account the nonlinear effects of the fracture process zone on DCB and ENF delamination testing. They concluded that applying this law the fracture behavior is properly determined. Huan and Li [30] proposed a novel and simple approach to predict initiation and propagation of delamination. Authors introduced a thin matrix-rich layer in the middle of two adjacent layers of unidirectional laminates. Then, using only G_Ic_ obtained from a DCB test, they determined the modification factor that should be applied to the stresses of the matrix layer. The way to predict delamination in the adjacent lamina is to analyze whether a tensile or shear failure occurs in the secondary layer, that is to say, the matrix-rich layer: if there is a failure, then delamination will occur. Giannis et al. [31] performed delamination testing on coupons of polymer composites used in the scope of rotor blades. The impact of the stiffness of the carbon fibers of different materials was analyzed on the value of fracture toughness using Mode-I fracture tests. Zarouchas et al. [32] investigated the behavior from a mechanical point of view of two I-beams using the delamination testing configuration of the four-point bending test. The I-beam represents a rescaled load-carrying blade structure. The Double Cantilever Beam (DCB) test for Mode-I was used by Shah and Tarfaoui [33], while the End Notched Flexure (ENF) test for Mode-II for characterizing bonded interfaces to determine which zones are the most critical in the wind turbine blade. Later, authors performed an experimental study on a sandwich specimen that was used in a real rotor blade [34]. They determined the energy release rates for both Mode-I and Mode-II. They carried out the DCB test to analyze Mode-I and the Single over Leg Bending test (SLB) to analyze Mode-II. The failure performance of composite materials in a substructure of a rotor blade was studied by Al-Khudairi and Ghasemnejad [35], using Kevlar, carbon and glass fibers. Wang and Soutis [36] carried out mechanical tests on a T-joint for wind turbine blade applications. With the experimental results, they validated an empirical model in order to predict the strength of composite T-joints. Beam type specimens made from adhesively-bonded fiber-reinforced composites was studied by Sørensen et al. [37] under steady-state Mode-I, Mode-II and mixed-mode loading conditions. An analytical approach for the Energy Release Rates (ERR) was deduced by authors, giving the determination of the fracture resistance of adhesive joints.

One of the biggest structures on a wind turbine blade is the spar cap and it is usually manufactured by means of unidirectional laminates. One of the major failures in the spar cap is the delamination. Besides, wind turbine blade bond lines are exposed to mixed-mode loading conditions, a combination of fracture Mode-I and Mode-II [38].

In this study, the End Notched Flexure test with Roller (ENFR) test method [39] is applied to the analysis of the mixed-mode I/II fracture in unidirectional composite materials that can be used in the spar cap of the wind turbine blade. This is a three-point bending test method. In this case, the specimen has a crack centered in the thickness, and in order to promote mixed-mode fracture, a metallic roller is inserted between the two arms of the crack. The main contribution of the ENFR test applied in this paper is that for the material analyzed and the geometry tested, with only one simple fracture test, the critical value of the energy release rate, which gives the fracture resistance of the material, can be obtained. Fifteen different test conditions are analyzed in order to assess the suitability of the method for the characterization of materials at coupon testing level, which is very important for the certification process of a wind turbine blade.

The remaining or the paper is organized as follows. In Section 2 authors briefly comment on theoretical aspects of delamination tests and fracture mechanics. In Section 3 the configuration of the delamination test used in this study and the analytical approach are presented, while Section 4 describes the test material, apparatus and procedure, the data reduction system and the crack-length determination procedure. Besides, the experimental results obtained from fifteen test conditions are depicted. Finally, the last section exposes the conclusions regarding the material toughness of this study.

## 2. Some Aspects on Fracture Mechanics and Delamination Tests

Laminates of composite materials are extensively used in rotor blades. They have the advantage that they can be designed depending on their application. However, the interlaminar fracture is one of their drawbacks. When an interlaminar crack or delamination appears, or adjacent plies separate, the composite material could fail, resulting on the failure of the blade structure.

**Figure 3:**
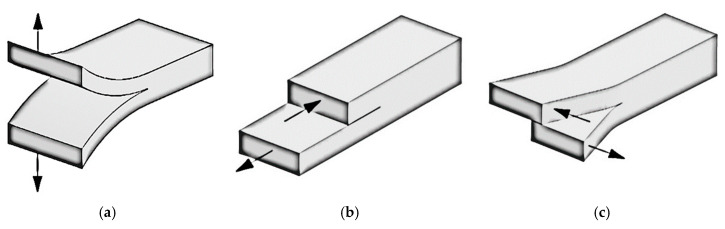
Modes of fracture: (**a**) Mode-I (opening); (**b**) Mode-II (sliding); (**c**) Mode-III (tearing) [42].

The Linear Elastic Fracture Mechanics (LEFM) theory is among the most used methods for crack analysis [40]. Delamination growth can be studied applying the energy criterion approach of fracture mechanics. In this study is assumed that if sufficient potential energy is available to get over the surface energy of the material, then a crack can grow. The energy release rate, denoted *G*, is the crack driving force. When *G* overcomes a critical value, *G_C_*, then the crack will grow. This critical value is the fracture toughness, and it is a material property [41]. There are three modes of fracture as it is shown in Figure 3.

The energy release rates (ERR) corresponding to each mode are *G_I_*, *G_II_* and *G_III_*, respectively. And the values of fracture toughness belong to each fracture mode are the following: *G_IC_*, *G_IIC_* and *G_IIIC_*. In the delamination tests, usually a load is applied in a beam type specimen with an artificial crack until the crack grows. The applied force and the displacement are obtained from the test machine. These are the input data for determining the crack length. Then the ERR can be determined by means of beam theory. Mixed-mode I/II is very common loading scenario in composite laminates [40].

## 3. Test Description

**Figure 4:**
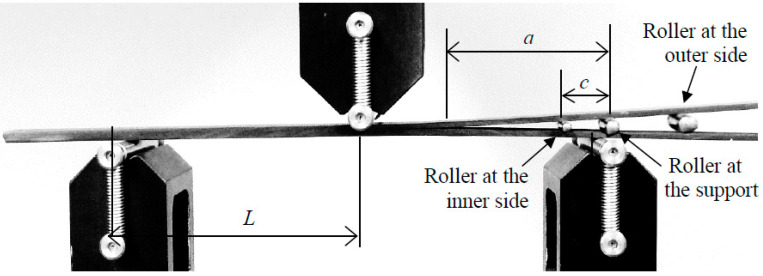
Test configuration showing the three positions of the roller [42].

The End Notched Flexure with inserted Roller (ENFR) [39] test configuration is applied. This test configuration is a three-point flexural test, and consists on applying the load to a beam type specimen with an artificially generated initial crack in the same direction than the fibers. The configuration test with three bending points is widely used in order to analyze fracture in composites. This configuration with an asymmetric beam was performed by Sayer et al. [43] for analyzing the adhesive performance between the spar cap and shear web of a rotor blade. The End Notched Flexure test (ENF) configuration is the one proposed by the ASTM for pure Mode-II delamination test. This configuration is also a three-point bending [44]. Using the ENFR test method, Mode-II is get by the applied load and the insertion of the roller between the two arms of the crack gives the opening mode or Mode-I. A sketch of the test is shown in Figure 4, where *a* is the crack length, *L* the half span of the test and *c* the distance from the roller position to the support. The metallic roller can be positioned at the inner side of the support, at the vertical of the support or at the outer side of the support.

A distinctiveness of this test is that when the roller is introduced, the load application point suffers a vertical displacement, an initial negative displacement called $\delta_{0}$ and therefore, the compliance of the test is expressed as:

(1) $$C=\frac{\delta -\delta_{0}}{P}$$

The compliance of the ENFR test for the three positions of the roller in terms of test parameters can be expressed as follows [39]:

(2) $$\begin{array}{c} Outer\;Side\;\;\;\;\;\;\;C=\frac{1}{8E_{F}b^{2}h^{3}}\;\left[\frac{{\left(a-c_{0}\right)}^{3}\left(3a^{3}+3ac_{0}^{2}+c_{0}^{3}\right)}{a^{3}}+2L^{3}\right]\;\\ Inner\;Side\;\;\;\;\;\;\;C=\frac{3a^{3}+2L^{3}+c_{i}^{3}+3ac_{i}^{2}}{8E_{F}b^{2}h^{3}}\;\;\;\;\;\\ c=0\;\;\;\;\;\;\;\;\;\;\;C=\frac{3a^{3}+2L^{3}}{8E_{F}b^{2}h^{3}} \end{array}$$

where *b* is the width of the specimen, and *h* is half the thickness.

Energy release rates as fracture mechanical characteristic features play a major role in the characterization of composites. In the ENFR test, the energy release rate, *G*, is determined based on the complementary strain energy as [39]:

(3) $$G=\frac{1}{b}{\left(\frac{\partial U^{*}}{\partial a}\right)}_{Fi_{cte}}$$

where U* is the complementary strain energy, *da* is the differential crack advance and *b* is the width of the specimen. Using beam theory for calculating bending moments, shear forces and the complementary strain energy, the ERR of each mode of fracture can be expressed as follows [39]:

(4) $$\begin{array}{cc} Outer\;Side\;G_{I}=\frac{3R^{2}E_{f}h^{3}}{4a^{4}}-\frac{3PRc_{0}\left(a^{2}-c_{0}{}^{2}\right)}{4a^{4}b}+\frac{3P^{2}c_{0}{}^{2}{\left(a^{2}-c_{0}{}^{2}\right)}^{2}}{16a^{4}E_{F}b^{2}h^{3}} & G_{II}=\frac{9P^{2}\left(a-c_{0}{}^{2}\right)}{16E_{F}b^{2}h^{3}}\\ Inner\;Side\;G_{I}=\frac{3R^{2}E_{f}h^{3}}{4{\left(a-c_{i}\right)}^{4}}-\frac{3PRc_{i}}{4b{\left(a-c_{i}\right)}^{2}}+\frac{3P^{2}c_{i}{}^{2}}{16E_{F}b^{2}h^{3}} & G_{II}=\frac{9P^{2}a^{2}}{16E_{F}b^{2}h^{3}}\\ c=0\;\;\;\;\;G_{I}=\frac{3R^{2}E_{f}h^{3}}{4a^{4}} & G_{II}=\frac{9P^{2}a^{2}}{16E_{F}b^{2}h^{3}} \end{array}$$

where outer side, inner side and *c* = 0 mean the three possible positions of the roller, see Figure 4. *G_I_* is the ERR part due to the opening fracture mode during the delamination advance, and *G_II_* is the part due to the shear fracture mode. A finite element method denominated two-step extension procedure was used to validate this analytical mode-partition [39].

As for mixed-mode fracture testing, finding a suitable failure criterion is a fundamental issue. In pure Mode-I the crack will grow when *G_I_* overcomes its critical value or fracture toughness, *G_IC_*, and in pure Mode-II when *G_II_* overcomes *G_IIC_*. Thus, the crack will grow when:

(5) $$\begin{array}{c} G_{I}>G_{Ic}\\ G_{II}>G_{IIc} \end{array}$$

Nevertheless, in mixed-mode fracture, even though the total ERR *G* = *G_I_* + *G_II_*, the fracture toughness *G_C_* ≠ *G_IC_* + *G_IIC_*. The procedure to determine a failure criterion lies on fitting test values for *G_I_* and *G_II_* obtained at a number of different mixed-mode ratios. Several failure criteria have been investigated for mixed-mode fracture [45,46]. One of the most widely used criterion to predict mixed-mode delamination is the linear criterion, which establishes an interaction between *G_I_* and *G_II_*, see Equation (6), and under this criterion the crack propagation will occur if the sum is greater than 1. For the ENFR test configuration, the linear failure criterion is taken as a preliminary hypothesis.

(6) $$\frac{G_{I}}{G_{Ic}}+\frac{G_{II}}{G_{IIc}}=1$$

Provided that the linear criterion is fulfilled, and expressing Equation (6) in a similar manner to Equation (5), the crack propagation condition can be expressed as follows:

(7) $$G_{I}G_{Ic}+G_{II}G_{IIc}>G_{Ic}G_{IIc}$$

The member on the left side is the energy available for crack growth and the member on the other side is the critical value for crack propagation. Using Equations (5) and (7) an equivalent energy release rate *G_eq_* can be expressed as follows:

(8) $$\begin{array}{c} G_{eq}={\left(G_{I}G_{Ic}+G_{II}G_{IIc}\right)}^{\frac{1}{2}}\\ G_{eqc}={\left(G_{Ic}G_{IIc}\right)}^{1/2} \end{array}$$

*G_eq_* is the equivalent energy release rate, and *G_eqc_* is defined as the critical value of the equivalent energy release rate. Accordingly, the condition for crack propagation under mixed-mode fracture in the ENFR test configuration is denoted in a simpler manner as:

(9) $$G_{eq}>G_{eqc}$$

## 4. Experimental Results

### 4.1. Materials and Test Apparatus

**Figure 5:**
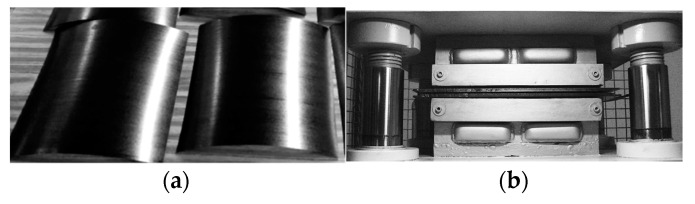
Manufacturing process of the test specimens: (**a**) Prepregs samples before stacking; (**b**) Closed-mold placed in the press

The unidirectional laminates of sixteen layers [0]_16_ used in this work were manufactured by T300/F593 prepregs and provided by Hexcel Composites (Madrid, Spain), with a specific volume-content of fiber of 55%. The carbon fiber was the T300 and F593 was the epoxy matrix. A prepreg is a reinforcing fabric, which has been pre-impregnated with a resin system in a pre-curing state. Exposing it to high temperatures under certain pressure, the prepreg transforms into a solid material, lightweight and stiff. The plates were manufactured by hot press molding. The initial crack was created placing a Teflon film in the middle step of the stacking process, in this case after the 8th layer of prepregs, Figure 5a. When the piling up process was finished, the mold was covered by a metallic plate and it was placed in the press, Figure 5b. The curing cycle defined by the manufacturer was then applied to perform the curing process.

**Figure 6:**
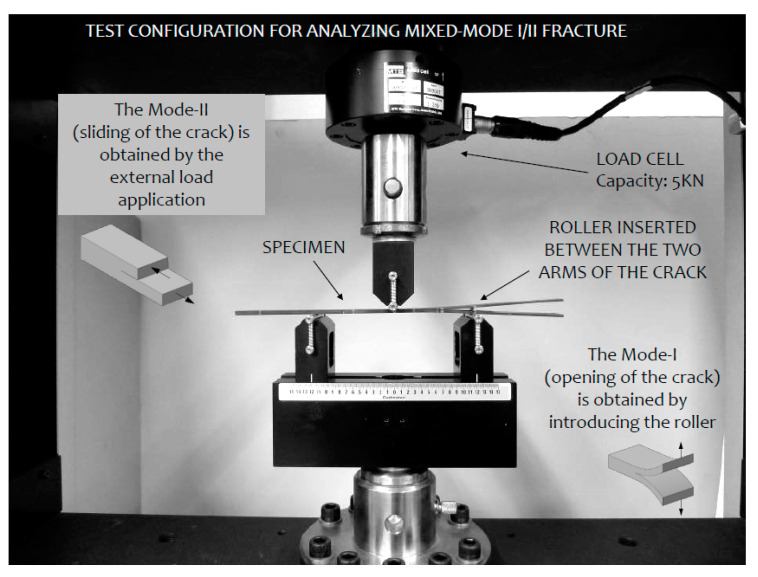
Lay out of the test. Specimen positioned in the test rig.

The specimens were cut by means of a diamond disc saw. The nominal dimensions of the specimens were 3 mm thickness, 15 mm width and 250 mm length, respectively. Tests were performed on an MTS-Insight 100 electromechanical testing machine (MTS Systems Corporation, Eden Prairie, MN, USA), see Figure 6. It was operated in a displacement-controlled mode while equipped with a 5 kN load cell.

Before starting fracture tests, it is necessary to determine the elastic moduli of each sample. They were determined based on a procedure proposed by Mujika [47]. These flexural tests were carried out in the zone without crack of the specimen at five nominal spans: 70, 80, 90, 100 and 120 mm. The results obtained were:

*E_f_* = 108.1 (±2.4) GPa

*G_LT’_* = 4.3 (±0.5) GPa

where *E_f_* is the flexural modulus and *G_LT’_* is the out of plane shear modulus. The velocity of the load application point is not always the same. It has been calculated in order to get a constant displacement rate of 0.01 min^−1^, which was the one proposed in the standard ISO 14125 [48]. The velocity of movement of the load application head had been determined from the displacement of the midpoint in the ENFR test [39]. Expressing the displacement in terms of the deformation and differentiating it with respect to time, the velocity of the load application in order to get a deformation rate of 0.01 min^−1^ could be obtained.

Every specimen had to be prepared propagating the artificial initial crack around 5 mm. This crack propagation was performed in Mode-II in the ENF test configuration in order to avoid the effect of the resin-rich area.

### 4.2. Test Methodology

**Figure 7:**
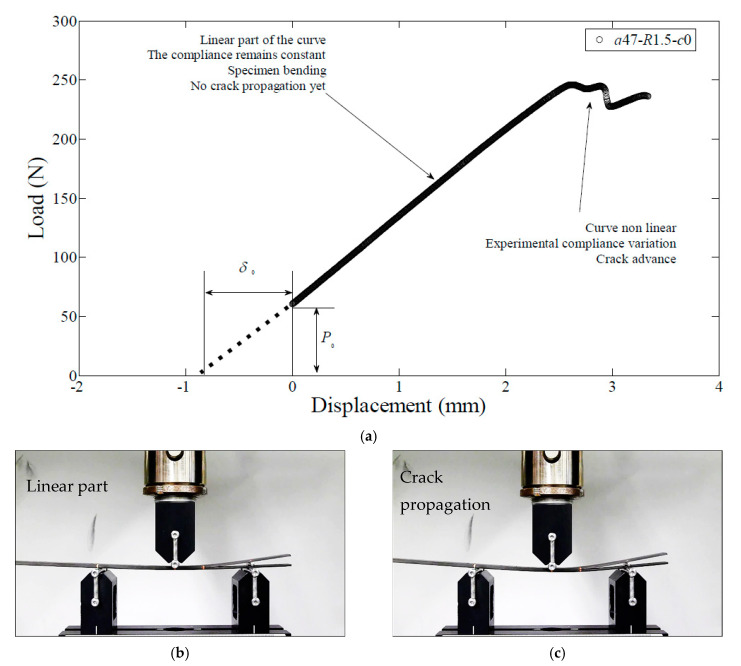
Test steps during and ENFR mixed-mode test: (**a**) Experimental load-displacement curve; (**b**) Photograph of a step of the linear part of the test; (**c**) Photograph of a step during crack propagation.

Provided that the specimen geometry and the material are defined previously, three parameters can be modified in the ENFR test configuration in order to get different contribution of *G_I_* and *G_II_*. These parameters are the roller position, the roller radius and the initial crack length. Based on this fact, *a*_i_-*R*_j_-*c*_k_ is the nomenclature that has been followed in this work being *a*_i_ the initial crack length; *R*_j_ the radius of the roller; and *c*_k_ the position of the roller. Once these three parameters were defined, the roller is collocated in the proper position and the specimen was collocated in the rig, as is shown in Figure 7a. As stated before, when the roller is introduced the load application point suffers a vertical negative displacement and therefore the load-displacement curve does not pass through the origin of coordinates.

As it is shown in Figure 7a there is an initial manual loading application descending the load application head until the displacement is zero before the test started. During the first part of the test, the load–displacement curve is linear and thence the experimental compliance remains constant. The specimen is suffering bending but the crack propagation has not started yet, as it can be seen in Figure 7b. When the crack starts to propagate at the initiation point, the load displacement curve lost its linearity, and there is a variation of the experimental compliance, Figure 7c.

**Figure 8:**
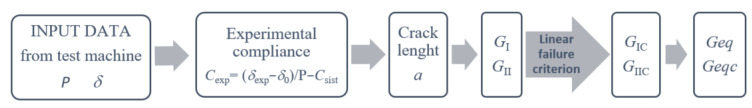
Data reduction scheme.

In order to get the fracture parameters, data reduction procedure is explained in the flow chart of Figure 8:

As shown in Figure 8 during each propagation test, the applied load and the application point displacement are measured by the test machine. Taking into account the stiffness of the test rig, the experimental compliance is then determined as:

(10) $$C_{spec}=\frac{\delta_{exp}-\delta_{0}}{P}-C_{sist}$$

where *C_spec_* is the experimental compliance of the specimen, *C_sist_* is the system compliance including the stiffness of the load application chain and *δ_exp_* the displacement measured by the test machine. The system compliance has been calculated in a previous test, and the value obtained was *C_sist_* = 1/24,000 mm/N.

The determination of the crack length is performed by means of an iterative process using MATLAB software (R2020b), seeking for the value of *a* that fulfils that theoretical compliance given in Equation (2) is equal to the experimental compliance of the specimen given in Equation (10). Thereupon, the crack length is determined at each point of the test where load and displacement are measured, without any optical device [49].

Following the flow diagram shown in Figure 8 and substituting the value of *a*, the value of the load measured in the test machine and the rest of the already known parameters in Equation (4), the ERR corresponding to each mode of fracture, *G_I_* and *G_II_*, are obtained at every point of the crack propagation where load and displacement have been measured. The next step is to verify that the linear criterion of Equation (6) is met in order to obtain the critical values of the energy release rates, and hence the equivalent energy release rate presented in Equation (8).

The relation between *G_I_* and *G_II_*, or the mode-mixity, is called the mode ratio in this study is taken as *G_II_/G*, where G is the total energy release rate and in this case was *G* = *G_I_* + *G_II_*.

The nominal dimensions of the test are width *b* = 15 mm, span 2*L* = 120 mm and thickness 2*h* = 3 mm. The initial mode ratio analyzed in this work is between 65% and 75% approximately, with a number of different testing conditions. All of the test conditions are summarized in Table 1.

### 4.3. Results and Discussion

As it is shown in Figure 8, the final output of the ENFR delamination test are *G_IC_*, *G_IIC_* and *G_eqc_*. Fracture toughness for Mode-I and Mode-II, that is, the values of *G_IC_* and *G_IIC_* are obtained after fitting the results of all the test to the linear failure criterion of Equation (6). The mean value and the standard deviation obtained are:

*G_IC_* = 273 (±32) J/m^2^

*G_IIC_* = 1177 (±179) J/m^2^

**Figure 9:**
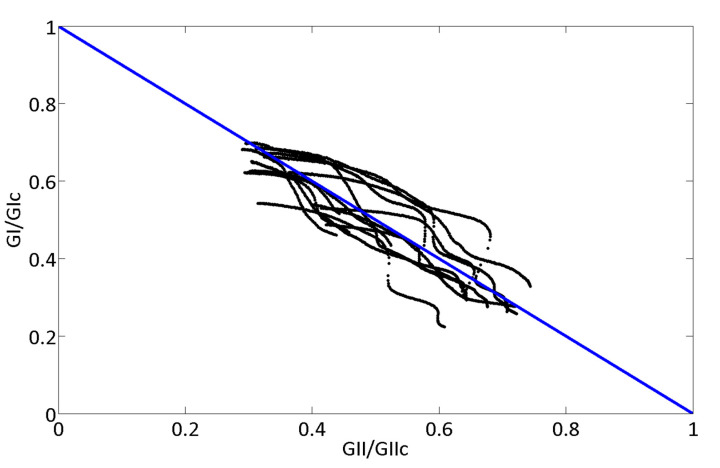
Fracture envelope.

These values agree with the ones obtained in pure mode tests [50,51] published before. The determination coefficient R^2^, gives the quality of the linear regression [52] and the mean value resulted in 0.89. In Figure 9, the fracture envelope is shown. The straight blue line represents the linear criterion that corresponds to the mean values of *G_IC_* and *G_IIC_* mentioned above. The black dots are all the experimental data of the fifteen test conditions presented in Table 1 during crack propagation. As it can be seen in Figure 9 they fit the linear criterion.

Substituting the values of *G_IC_* = 273 (±32) J/m^2^ and *G_IIC_*= 1177 (±179) J/m^2^ in Equation (8), the critical value of the theoretical equivalent energy release rate was *G_eqc_* = 567 (J/m^2^).

**Figure 10:**
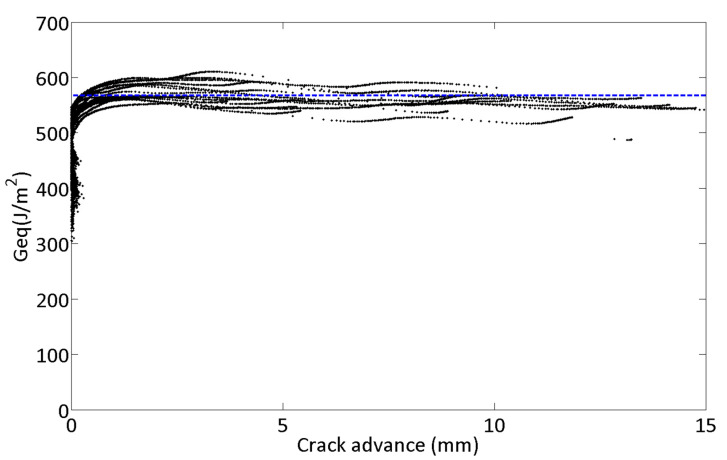
Experimental values of G_eq_ during crack propagation for all the tests.

Since the linear criterion is suitable for representing the mixed-mode fracture of the material of this work on the ENFR test configuration, *G_eq_* must be assessed. According to the mixed-mode fracture crack propagation condition from Equation (7), if the experimental value of the equivalent ERR tends to a constant value during crack propagation, that value can be considered the critical value of *G_eq_*, thus, it can be considered a material property, i.e., the mixed-mode fracture toughness. In Figure 10 the values of *G_eq_* for all tests are depicted. The line in blue represents the theoretical value of *G_eqc_* = 567 (J/m^2^).

As it can be seen in Figure 10, the experimental values of *G_eq_* present a plateau. Furthermore, the mean value of all the data from a crack advance of Δ*a* = 0.5 mm was 568 ± 19 J/m^2^. The relative error between that mean value and the theoretical value of *G_eqc_* was less than 1%. Consequently, the equivalent energy release rate approach can be considered suitable to represent the fracture criterion for the material studied, and the critical value can be considered a material property.

**Figure 11:**
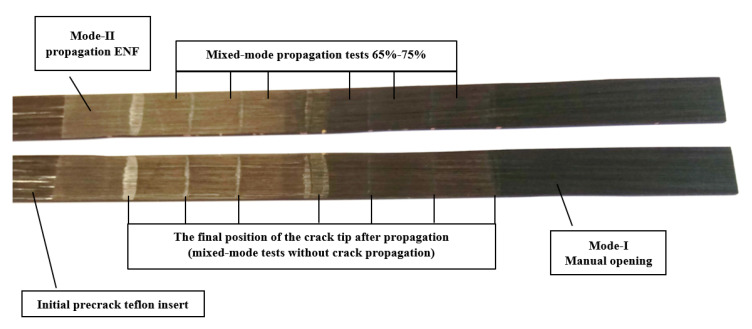
Specimen open for micrography examination.

Besides, microstructural observation of the fracture surfaces was carried out by optical microscopy. The surface morphology was examined at the nanoscale using an scanning electron microscopy. Prior to examination, all the samples were cleaned with ethanol to eliminate impurities such as dust. Then in order to improve the conductivity by the electrons absorbed during the SEM observation, a thin layer of gold was applied to the fracture surfaces. Four different fracture types have been examined. In Figure 11 one specimen opened by hand after finishing all the tests is shown, to determine the degree to which each failure mode contributed. Starting from the left, the bright part corresponds to the artificial initial crack made by Teflon film. The next part corresponds to the first crack propagation in pure Mode-II, in order to avoid the effect of the resin-rich area as stated before. The following parts corresponds to crack propagation in mixed-mode at different mode ratios. Finally, the last part on the right of the specimen is supposed to be fracture surface corresponding to Mode-I, because the specimen have been opened by hand. It is not possible to ensure pure Mode-I due to the manually opening. The vertical lines are the final position of the crack tip after propagation, In other words the separation between one propagation test and the following one. The different widths of these vertical lines are due to the fact that between one propagation test and the next one, some test without crack propagation were performed, in order to check the initial crack tip position.

**Figure 12:**
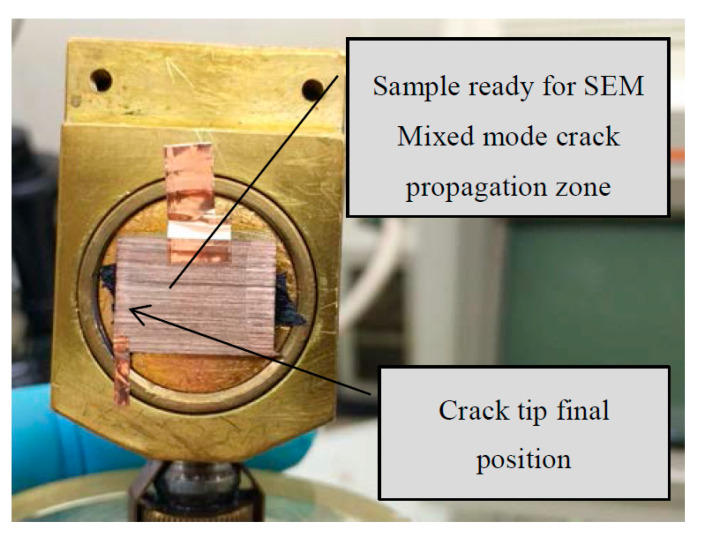
Sample prepared for scanning electron microscope.

In Figure 12 one section of the specimen prepared for the scanning optical microscopy is shown. In this case the section that can be seen corresponds to mixed-mode fracture.

**Figure 13:**
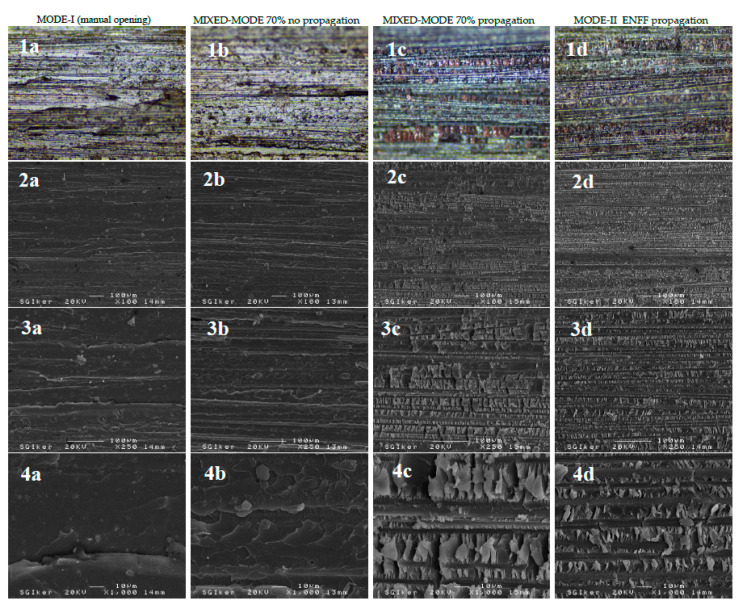
Optical and SEM micrographs. First row: optical ×10, Second row: SEM ×100, Third row SEM ×250 Forth row SEM ×1000. (**a**) Mode-I, manual opening (**b**) Mixed-mode tests without propagation (**c**) Mixed-mode tests with crack propagation (**d**) Mode-II test with crack propagation.

In Figure 13 optical micrographs and SEM micrographs for the four types of fracture are shown. The first row corresponds to the optical microscope and the second, third and fourth rows are the SEM micrographs.

Micrographs of the same row are taken at the same magnification. Micrographs of the same column correspond to the same type of surface.

As it can be seen in Figure 13 fracture surface corresponding to Mode-I, the one in the first column, is smoother than the others. As the contribution of the Mode-II increases, surface roughness is much greater and there can be seen chunks of resin [53]. The part corresponding to mixed-mode test without crack propagation has similar appearance as the Mode-I fracture surface.

## 5. Summary and Conclusions

Wind turbine blades are usually manufactured from fiber-reinforced composites. The trustworthy determination of composites improves the efficiency of wind turbines. Fracture damage in composite materials may drastically reduce the component performance. A mixed-mode test configuration, the End Notched Flexure test with Roller (ENFR), was applied for fracture mechanics characterization of composites. The equivalent energy release rate was assessed during crack propagation. The experimental data obtained agreed with the theoretical value, being the method valid for characterizing mixed-mode fracture. The analyzed delamination test can be used for obtaining the value of *G_eqc_* with one simple mixed-mode test configuration, provided that the linear criterion is fulfilled. The fracture surfaces have been explored using optical microscopy and scanning electron microscopy finding different features depending on the fracture mode mixity. Therefore, at the coupon testing level and with the aim of characterizing unidirectional composite laminates of wind turbine, this procedure can be considered as an alternative.

## Figures and Tables

**Table 1:** Summary of the test parameters for the fifteen cases.

Test Number	Crack Length *a* (mm)	Roller Radius *R* (mm)	Roller Position *c* (mm)	Initial *G_II_*/*G* (%)
1	40	1	5	65
2	42	0.9	8	65
3	43	0.9	8	66
4	41	1	5	66
5	42	1.5	0	67
6	43	0.9	8	67
7	41	1	5	68
8	42	1.5	0	68
9	45	1	8	68
10	40	1.25	0	71
11	43	1.5	0	72
12	45	1.5	0	74
13	46	1.5	0	77
14	47	1.5	0	77
15	40	0.5	10	79

## Data Availability

Data sharing not applicable. No new data were created or analyzed in this study. Data sharing is not applicable to this article.
